# 15d-PGJ_2_ Promotes ROS-Dependent Activation of MAPK-Induced Early Apoptosis in Osteosarcoma Cell In Vitro and in an Ex Ovo CAM Assay

**DOI:** 10.3390/ijms222111760

**Published:** 2021-10-29

**Authors:** Mateja Mikulčić, Nassim Ghaffari Tabrizi-Wizsy, Eva M. Bernhart, Martin Asslaber, Christopher Trummer, Werner Windischhofer, Wolfgang Sattler, Ernst Malle, Andelko Hrzenjak

**Affiliations:** 1 Department of Internal Medicine, Division of Pulmonology, Medical University of Graz, 8036 Graz, Austria; mateja.mikulcic@medunigraz.at; 2 Otto Loewi Research Center, Division of Immunology and Pathophysiology, Medical University of Graz, 8010 Graz, Austria; nassim.ghaffari@medunigraz.at; 3 Gottfried Schatz Research Center, Division of Molecular Biology and Biochemistry, Medical University of Graz, 8010 Graz, Austria; eva.bernhart@medunigraz.at (E.M.B.); christopher.trummer@medunigraz.at (C.T.); wolfgang.sattler@medunigraz.at (W.S.); ernst.malle@medunigraz.at (E.M.); 4 Diagnostic and Research Institute of Pathology, Medical University of Graz, 8010 Graz, Austria; martin.asslaber@medunigraz.at; 5 Department of Pediatrics and Adolescence Medicine, Medical University of Graz, 8036 Graz, Austria; werner.windischhofer@medunigraz.at; 6 Ludwig Boltzmann Institute for Lung Vascular Research, Medical University of Graz, 8010 Graz, Austria

**Keywords:** 15d-PGJ_2_, apoptosis, CAM assay, osteosarcoma, reactive oxygen species

## Abstract

Osteosarcoma (OS) is the most common type of bone tumor, and has limited therapy options. 15-Deoxy-Δ^12,14^-prostaglandin J_2_ (15d-PGJ_2_) has striking anti-tumor effects in various tumors. Here, we investigated molecular mechanisms that mediate anti-tumor effects of 15d-PGJ_2_ in different OS cell lines. Human U2-OS and Saos-2 cells were treated with 15d-PGJ_2_ and cell survival was measured by MTT assay. Cell proliferation and motility were investigated by scratch assay, the tumorigenic capacity by colony forming assay. Intracellular ROS was estimated by H_2_DCFDA. Activation of MAPKs and cytoprotective proteins was detected by immunoblotting. Apoptosis was detected by immunoblotting and Annexin V/PI staining. The ex ovo CAM model was used to study growth capability of grafted 15d-PGJ_2_-treated OS cells, followed by immunohistochemistry with hematoxylin/eosin and Ki-67. 15d-PGJ_2_ substantially decreased cell viability, colony formation and wound closure capability of OS cells. Non-malignant human osteoblast was less affected by 15d-PGJ_2_. 15d-PGJ_2_ induced rapid intracellular ROS production and time-dependent activation of MAPKs (pERK1/2, pJNK and pp38). Tempol efficiently inhibited 15d-PGJ_2_-induced ERK1/2 activation, while N-acetylcystein and pyrrolidine dithiocarbamate were less effective. Early but weak activation of cytoprotective proteins was overrun by induction of apoptosis. A structural analogue, 9,10-dihydro-15d-PGJ_2_, did not show toxic effects in OS cells. In the CAM model, we grafted OS tumors with U2-OS, Saos-2 and MG-63 cells. 15d-PGJ_2_ treatment resulted in significant growth inhibition, diminished tumor tissue density, and reduced tumor cell proliferation for all cell lines. Our in vitro and CAM data suggest 15d-PGJ_2_ as a promising natural compound to interfere with OS tumor growth.

## 1. Introduction

Osteosarcoma (OS), a tumor of mesenchymal origin, represents the most common primary malignancy of bones and the most frequent malignant bone tumor [1,2]. OS occurs predominantly in adolescents and is the eighth most common pediatric cancer. Despite well-established therapy protocols, patients suffering from this high-grade malignant tumor have a very poor survival rate. Patients with localized disease have a 5-year overall survival of 60–70% [3,4,5], however, recurrences are frequent and develop in 40–50% of patients during treatment [6,7]. Beside surgery, neoadjuvant therapy based on combination of methotrexate-doxorubicin-cisplatin is established, albeit very aggressive and toxic [8]. Therefore, not only optimization of current therapy protocols, but also discovery of alternative strategies and development of novel therapeutic approaches are required. Currently, several ongoing clinical trials are evaluating treatment strategies to improve the unfavorable prognosis for high-grade osteosarcoma patients undergoing conventional treatment [9,10]. Thus, a better understanding of molecular mechanisms involved in OS tumorigenesis is essential.

Besides genetic alterations [4], etiological factors such as cyclooxygenase and its respective metabolites, the prostaglandins (PGs), are pivotal bioactive molecules involved in bone resorption [11] and bone formation [12,13], but also in bone patho/physiology and tumorigenesis. PGE_2_, the most abundant PG species synthesized by various solid tumors, acts as a potent modulator of tumor cell proliferation, angiogenesis, invasion and immunosuppression [14]. However, PGD_2_ has also been shown to modulate osteoclast and osteoblast function in vivo and in vitro [11,15]. PGD_2_ is highly unstable under in vivo and in vitro conditions, although reported to contribute to cellular migration, apoptosis, and matrix calcification of bone tissue [16,17]. Interestingly, the final but stable degradation product of PGD_2_, 15-deoxy-Δ^12,14^-PGJ_2_ (15d-PGJ_2_), has been shown to regulate a variety of cellular events ranging from cell growth to apoptosis [18]. These characteristics of 15d-PGJ_2_ are based on its electrophilic character as the α,β-unsaturated ketone moiety may form covalent protein adducts via Michael addition with cellular nucleophiles, preferentially thiol groups. Under basal conditions, picomolar to nanomolar concentrations (and even higher) of free 15d-PGJ_2_ have been measured in biological fluids [19], whereas increased cyclic PG levels have been reported during late stages of inflammation and cancer [20]. Effects of 15d-PGJ_2_ have been reported in different cellular systems. Cytotoxic effects of 15d-PGJ_2_ have been described in uterine cancer [21], breast cancer [22], prostate cancer [23], colon cancer [24], and to a certain extent also in OS cells [25,26]. Our group showed that in MG-63 OS cells, these cytotoxic effects are based on the activation of the mitogen-activated protein kinase (MAPK)/Akt axis [25], whereas Yen and coworkers showed that 15d-PGJ_2_ induces reactive oxygen species (ROS)-mediated Akt inhibition and cell cycle alterations [26]. However, the acute effects of 15d-PGJ_2_ and its molecular mechanism in other OS cells are still not described.

In the present study, we demonstrated that 15d-PGJ_2_ has pronounced cytotoxic effects in U2-OS and Saos-2 OS cell lines. These effects were mainly based on ROS-mediated apoptosis and were more pronounced in OS cell lines as compared to a non-malignant osteoblast cell line. Furthermore, we showed substantial inhibitory effects of 15d-PGJ_2_ on OS-tumor growth by employing the avian ex ovo chorioallantoic membrane (CAM) assay, providing an important step towards preclinical in vivo models that meet 3R requirements and allow high throughput development of new treatment modalities.

## 2. Results

### 2.1. 15d-PGJ_2_ Inhibits the Cell Growth, Colony Formation, and Motility of Human OS Cells

**Figure 1:**
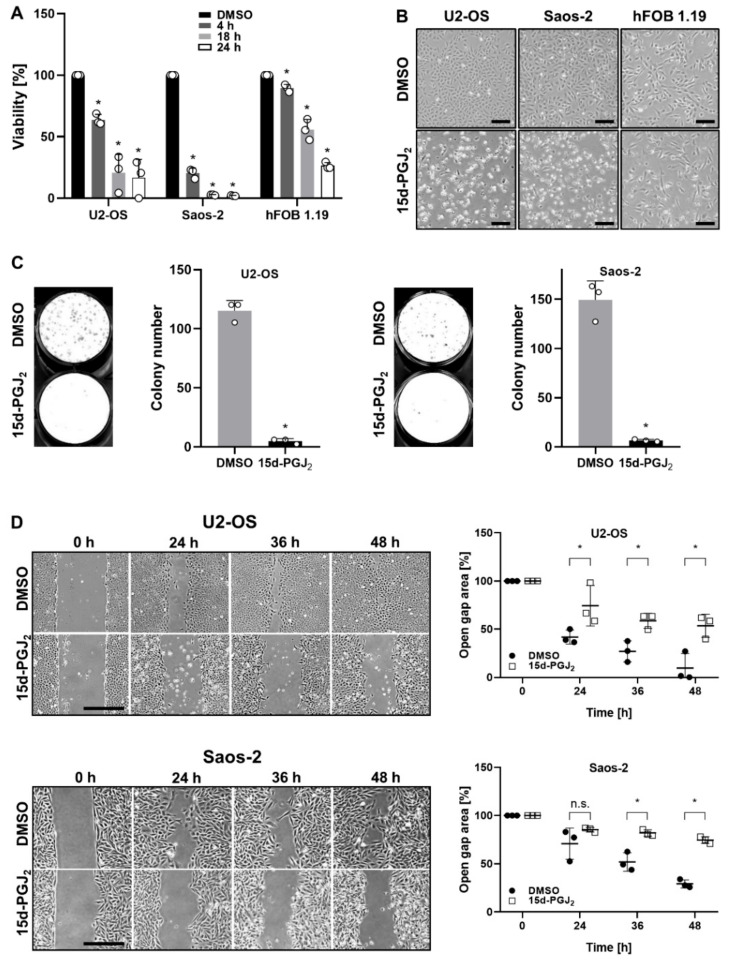
15d-PGJ_2_ inhibits cell growth, colony forming ability and motility of OS cell lines. (**A**) Human OS cell lines (U2-OS and Saos-2) and the human non-malignant osteoblastic cell line hFOB 1.19 were treated with 20 µM 15d-PGJ_2_ for indicated time periods. Cell viability was measured by MTT proliferation assay. DMSO concentration corresponding to the 20 µM 15d-PGJ_2_ (0.04%) was used as a vehicle control. Data are represented as means of three independent experiments and viability of DMSO-treated cells was set to 100% (* *p* ≤ 0.05). (**B**) Representative images display the change in morphology of OS and hFOB 1.19 cell lines observed upon treatment with 20 µM 15d-PGJ_2_ for 24 h. (scale bar = 100 µm). (**C**) Representative images (left panel) and bar graphs (right panel) out of three experiments show significant differences between the number of colonies formed by U2-OS and Saos-2 cells after a 10 and 15 day incubation, respectively. Both cell lines were treated for 24 h with 20 µM 15d-PGJ_2_ dissolved in DMSO. DMSO (0.04%) was used as vehicle control. (**D**) U2-OS (upper panel) and Saos-2 OS (lower panel) cells were seeded into 12-well plates containing cell culture inserts with a 500 µm cell-free gap and grown until approx. 90% confluence before 24 h treatment with 15d-PGJ_2_ or DMSO (0.04%) as vehicle control. Inserts were removed and the gap closure was measured and quantified at indicated time periods. Representative images and dot graphs for each cell line (performed in triplicates) are shown. Scale bar = 500 µm. The dot graphs display the differences in scratch closure at each time point, with zero time point set to 100%. Data are represented as means ± SD. (* *p* ≤ 0.05; n.s. = not significant).

U2-OS and Saos-2 cells were treated with 20 µM 15d-PGJ_2_, based on data published in our previous study [25]. Experiments with a human osteoblastic cell line (hFOB1.19) were included to figure out any difference between non-malignant and OS cell lines in response to 15d-PGJ_2_. As shown in Figure 1A, both U2-OS and Saos-2 OS cell lines showed significantly reduced viability already 4 h after the treatment by 37% (U2-OS) and 80% (Saos-2), respectively. This reduction was even more pronounced after 24 h, with 16% (U2-OS) and 2% (Saos-2) remaining cell viability. Cell viability of non-malignant human osteoblastic hFOB1.19 cells was reduced by 10% (4 h) and by 75% (24 h) following treatment with 15d-PGJ_2_. Summarized, non-malignant osteoblast cells showed a substantially milder response towards 15d-PGJ_2_ treatment when compared to both OS cell lines and cell viability was decreased in the following order: Saos-2 >> U2-OS > hFOB1.19. These results were supported by morphological changes in all three cell lines, hFOB1.19 cells showing the least pronounced effect in response to 15d-PGJ_2_ (Figure 1B).

The colony-formation assay was performed to further underscore the malignant potential of Saos-2 and U2-OS cell lines after treatment with 15d-PGJ_2_. Figure 1C shows a strongly diminished potential for colony formation of both OS cell lines in response to 24 h 15d-PGJ_2_ treatment. Upon 15d-PGJ_2_ treatment, only few OS cells survived being able to form colonies, which is indicative for a pronounced anti-tumorigenic effect of 15d-PGJ_2_ towards OS cells.

In order to investigate the influence of 15d-PGJ_2_ on proliferation and motility of U2-OS and Saos-2 cells, a wound healing (scratch) assay was performed. Our data showed significant differences in wound closure between vehicle-treated and 15d-PGJ_2_-treated OS cells, these differences being more pronounced in U2-OS as compared to Saos-2 cells (Figure 1D). These effects were based predominantly on diminished cell proliferation upon 15d-PGJ_2_ treatment and, at least partially, on altered cell motility. In vehicle-treated OS cell lines, amorphous scratch borders with outgrowing cells were observed over time. In contrast, in 15d-PGJ_2_-treated samples scratch borders remained smooth during the whole observation period (48 h), indicating altered cell motility.

### 2.2. 15d-PGJ_2_-Induced ROS Production in OS Cells Can Be Inhibited by Specific ROS Inhibitors

**Figure 2:**
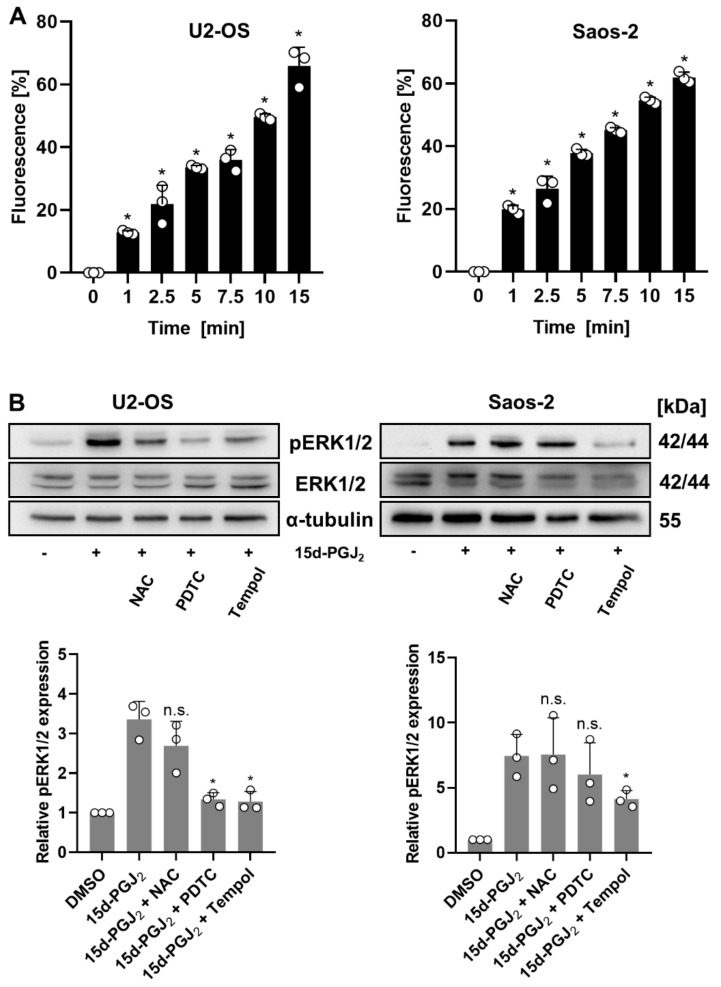
Induction of ROS production in OS cells treated with 15d-PGJ_2_. (**A**) Upon treatment of U2-OS (**left** panel) and Saos-2 (**right** panel) with 15d-PGJ_2_ for indicated time periods, OS cells were incubated with the ROS-reactive carboxy-H_2_DCFDA (5-(and -6)-carboxy-2′,7′-dichlorodihydrofluorescein diacetate) dye. The resulting changes in fluorescence were measured and displayed as bar graph for each cell line. The data are represented as means ± SD (* *p* ≤ 0.05) from three independent experiments. (**B**) Following a 30 min pre-incubation with ROS inhibitors (NAC (N-acetyl-cysteine), 15 µM; PDTC (pyrrolidine dithiocarbamate), 10 µM; Tempol, 10 µM), U2-OS (**left** panel) and Saos-2 (**right** panel) cells were exposed to the 20 µM 15d-PGJ_2_ for 15 min, followed by cell harvesting and lysis. The effects of the ROS inhibition on 15d-PGJ_2_-mediated intracellular MAPK activation (pERK1/2) were analyzed by immunoblotting experiments. One representative blot out of three is shown. Alpha-tubulin (α-tubulin) was used as a loading control (upper panel). Densitometric evaluation (lower panel) was based on at least three independent experiments and put in correlation to pERK1/2 expression in 15d-PGJ_2_-treated cells. Means ± SD are shown. (* *p* ≤ 0.05; n.s. = not significant).

As previously reported by our group [25], 15d-PGJ_2_ induces ROS production in MG-63 OS cells. Here, we wanted to investigate whether 15d-PGJ_2_-induced ROS activation is an early event and a characteristic feature of U2-OS and Saos-2 OS cell lines. Our results indicate a fast, statistically significant, and time-dependent ROS generation at similar extent in both cell lines (Figure 2A). In order to assess the importance of the augmented ROS production to further downstream signaling events triggered by 15d-PGJ_2_, we checked the status of phosphorylation/activation of extracellular signal-regulated kinase (ERK1/2, also known as p42/44 MAPK), an event commonly mediated by an increase in ROS levels. We detected strong phosphorylation of ERK1/2 in both cell lines already 15 min upon treatment (Figure 2B). We further used three different ROS scavengers (NAC (N-acetyl-cysteine); PDTC (pyrrolidine dithiocarbamate), and Tempol) to interfere with phosphorylation of ERK1/2 (Figure 2B). Preincubation with NAC (a cell permeable thiol that restores intracellular glutathione) slightly decreased ERK1/2 phosphorylation in U2-OS but not Saos-2 cells. The metal chelating antioxidant compound PDTC reduced ERK1/2 phosphorylation in U2-OS cells but was without significant effect in Saos-2 cells. Tempol, a superoxide dismutase (SOD) mimetic and superoxide anion radical scavenger, effectively attenuated ERK1/2 activation in both cell lines. These results suggest the involvement of 15d-PGJ_2_-induced ROS generation as an initiating event of ERK1/2 activation and subsequent induction of apoptosis.

### 2.3. 15d-PGJ_2_ Induces Acute Apoptosis in OS Cells

**Figure 3:**
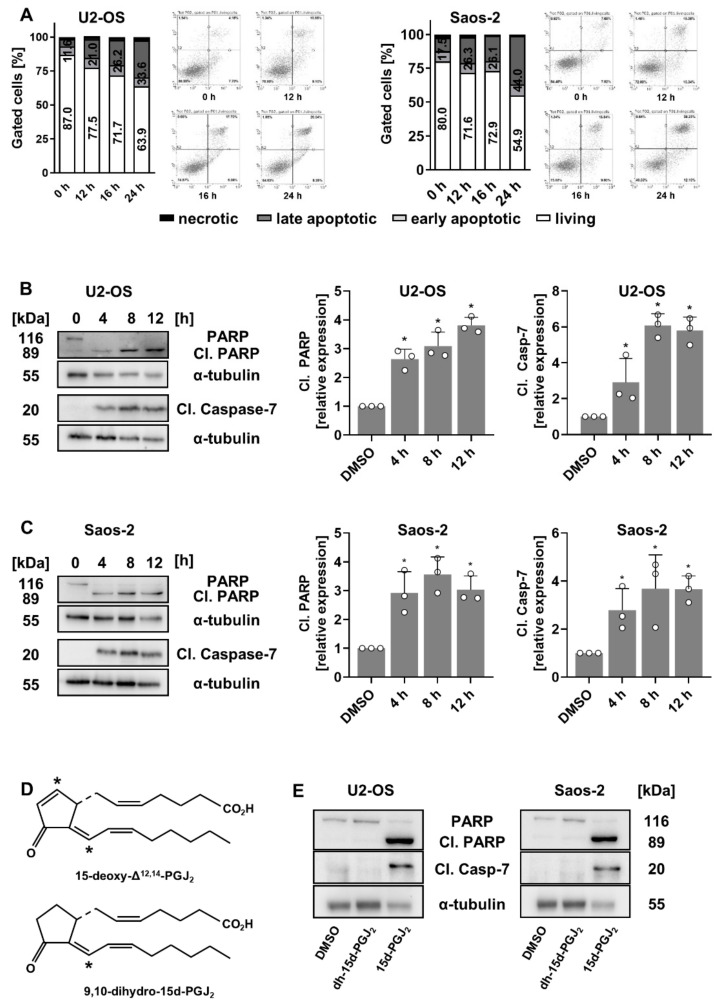
Induction of apoptosis in 15d-PGJ_2_-treated OS cell lines. (**A**) Following treatment of U2-OS (**left** panel) and Saos-2 cells (**right** panel) with 20 µM 15d-PGJ_2_ for indicated time periods, OS cells were double-stained for Annexin V/PI and analyzed by FACS. Results are displayed in bar graphs and scatter plots for both OS cell lines. Numbers indicate percentage of viable cells (white bars) and cells in early and late apoptosis (sum of both light and dark gray bars) compared to all gated cells set to 100%. One representative scatter plot from three independent experiments, each performed in triplicates, is shown. (**B**,**C**) U2-OS (**B**) and Saos-2 cells (**C**) were treated with 15d-PGJ_2_ for indicated time periods. Afterwards, cells were harvested, lysed and the expression of cleaved PARP (Cl. PARP) and cleaved pro-caspase-7 (Cl. Caspase-7) was assessed from total protein lysates by immunoblotting. One representative blot with corresponding densitometric evaluation based on at least three blots are shown. Alpha-tubulin (α-tubulin) was used as a loading control. The data are compared to time point zero and represented as means ± SD. (* *p* ≤ 0.05) (**D**) Structural formula of 15d-PGJ_2_ and 9,10-dh-15d-PGJ_2_, the structural analogue lacking the electrophilic carbon atom C9. (**E**) U2-OS (**left** panel) and Saos-2 OS cells (**right** panel) were treated with 20 µM 15d-PGJ_2_ or 9,10-dh-15d-PGJ_2_ for 18 h and apoptosis markers (Cl. PARP and Cl. pro-caspase-7) were analyzed by immunoblotting. One representative blot out of three is shown. Alpha-tubulin (α-tubulin) was used as a loading control.

We hypothesized that 15d-PGJ_2_ causes not only diminished cell proliferation, but also induction of apoptosis in OS cell lines. Thus, we performed Annexin V/PI staining and immunoblotting for the apoptotic marker proteins such as active caspase-7 and poly(ADP-ribose)-polymerase (PARP). The Annexin V/PI data indicated time-dependent activation in OS cell lines upon 15d-PGJ_2_ treatment (Figure 3A). Activation of apoptosis was slightly stronger in Saos-2 cells, this being in line with viability data (Figure 1A) and indicating higher sensitivity of Saos-2 towards 15d-PGJ_2_ compared to U2-OS cells. Even in less sensitive U2-OS cells, we observed robust and fast activation of apoptosis, with 21% and 33.6% of apoptotic cells after a 12 and 24 h treatment, respectively. The percentage of cell in early apoptosis was quite constant in range from 6% to 8% for both cell lines, whereas the percentage of necrotic cells was insignificant in all samples (below 1.5%). In Saos-2 cells, we detected 26.3% of apoptotic cells (in early and late apoptosis) at 12 h, and 44% at 24 h of 15d-PGJ_2_ treatment. This was further supported by a clear time-dependent caspase-7 and PARP cleavage in response to 15d-PGJ_2_ (Figure 3B,C). Altogether, our data indicate strong and early activation of apoptosis in both 15d-PGJ_2_-treated OS cell lines.

Previously, we showed that 9,10-dihydro-15d-PGJ_2_ (dh-15d-PGJ_2_), the structural analogue of 15d-PGJ_2_, did not induce ROS-mediated cell death in MG-63 OS cells [25,27]. To test whether the 9,10 double bond in 15d-PGJ_2_ is a structural prerequisite (Figure 3C) for its cytotoxic properties in OS cell lines, we compared activation of apoptosis in U2-OS and Saos-2 cells treated with either 15d-PGJ_2_ or dh-15d-PGJ_2_. As shown in Figure 3D, dh-15d-PGJ_2_ did not induce apoptosis in OS cells, indicating the functional importance of the cyclopentenone structure as an electrophilic mediator of cytotoxic cellular responses. These data show that the electrophilic carbon atom of 15d-PGJ_2_ is a prerequisite for 15d-PGJ_2_-based cell toxicity and support previous findings obtained in osteoclasts and MG-63 OS cells [17,25].

### 2.4. 15d-PGJ_2_ Induces Time-Dependent MAPK Activation in OS Cells

**Figure 4:**
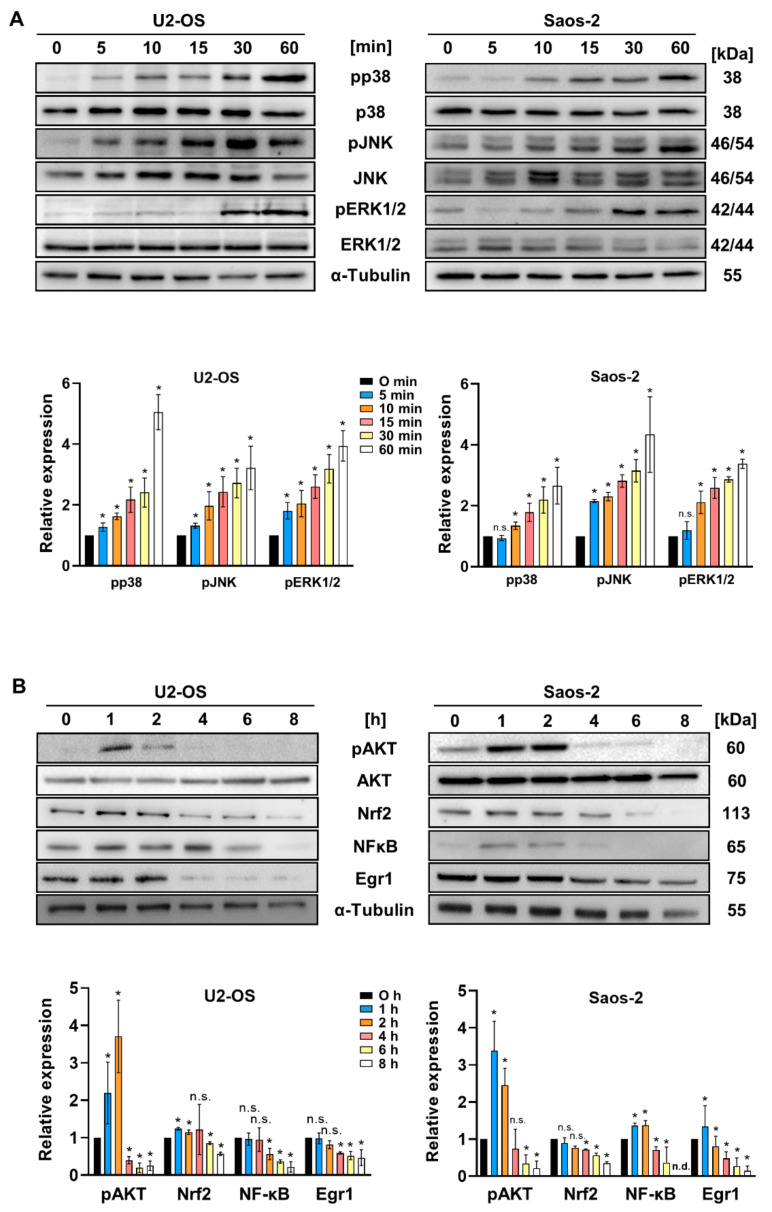
Activation of the MAPK pathway as an early downstream target of 15d-PGJ_2_. (**A**) U2-OS (**left** panel) and Saos-2 OS cells (**right** panel) were treated with 20 µM 15d-PGJ_2_ for indicated time periods and time-dependent MAPK phosphorylation status was analyzed by immunoblotting. One representative blot with corresponding densitometric evaluation based on at least three experiments are shown below. Alpha-tubulin (α-tubulin) was used as a loading control. The data represented as means ± SD are compared to time point zero. (* *p* ≤ 0.05; n.s. = not significant) (**B**) In order to assess whether the further downstream effects of 15d-PGJ_2_ include any cyto-protective mechanisms, immunoblots for antioxidant, pro-survival enzymes (AKT, Nrf2, NF-κB, Egr1) were performed. One representative blot and densitometric evaluation of three independent experiments are shown (below). Alpha-tubulin (α-tubulin) was used as a loading control. The data represented as means ± SD are compared to time point zero. (* *p* ≤ 0.05; n.s. = not significant); n.d. = not detected.

MAPK signaling cascades are activated in response to environmental stress signals and integrate cell fate decisions to induce survival or death pathways [28]. We aimed to analyze the effect of 15d-PGJ_2_ on the expression of MAPKs. Following the addition of 15d-PGJ_2_ we noticed a significant time-dependent increase in the phosphorylation/activation of the three members of the MAPK family, namely ERK1/2, p38 MAPK, and c-Jun N-terminal kinase (JNK) in both OS cell lines (Figure 4A). Next, we wanted to figure out whether these events have any effect on some of the pro-survival proteins as well as on pro-apoptosis intracellular protein responses. Therefore, OS cells were treated up to 8 h in order to identify changes in the phosphorylation of AKT, and expression of the basic leucine zipper transcription factor nuclear factor E2-related factor 2 (Nrf2), the zinc-finger transcription factor early growth response factor (Egr1), and nuclear factor NF-kappa B (NF-κB), respectively. We noticed a transient increase in AKT phosphorylation at 1 and 2 h that was significantly downregulated at later time points. Nrf2 and NF-κB expression was also induced at the earlier time points (up to 2–4 h), while Egr1 started to decrease at incubation times > 2 h (Figure 4B).

### 2.5. 15d-PGJ_2_ Inhibits OS Tumor Growth in the CAM Model

**Figure 5:**
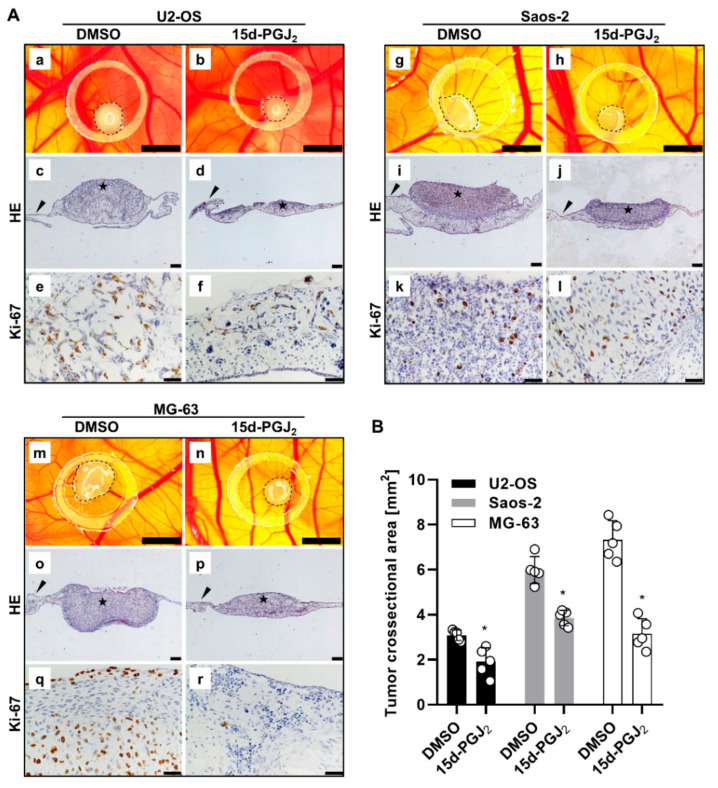
15d-PGJ_2_ reduces cell proliferation in OS cells grafted ex ovo on chicken chorioallantoic membrane (CAM). (**A**) Representative images display U2-OS, Saos-2, and MG-63 OS xenografts treated with vehicle control (DMSO concentration corresponding to 20 µM 15d-PGJ_2_, 0.04%; n = 5) or with 20 µM 15d-PGJ_2_ (n = 5). Microphotographs display differences in solid tumor formation for control (DMSO) and 15d-PGJ_2_-treated OS cells (U2-OS (**a**,**b**); Saos-2 (**g**,**h**); MG-63 (**m**,**n**)). Xenografts were photographed through a stereo microscope directly on cam (scale bar = 2 mm). Haematoxylin-eosin (HE) staining of cross-sections of the xenografts display the inhibiting effect of 15d-PGJ_2_ on xenograft growth (U2-OS (**c**,**d**); Saos-2 (**i**,**j**); MG-63 (**o**,**p**)). Arrowheads are indicative for the CAM tissue, stars for the OS xenografts (scale bar = 200 µm). Immunohistochemical staining of the OS xenografts with anti Ki-67 antibody (U2-OS (**e**,**f**); Saos-2 (**k**,**l**); MG-63 (**q**,**r**)) shows the proliferation of OS cells on the CAM (scale bar = 20 µm). (**B**) Tumor size (crossectional area) was quantified by ImageJ. Data represent mean values (mm^2^) and SD of five different tumors. Significance was estimated by *t*-test. (* *p* ≤ 0.05).

**Figure 6:**
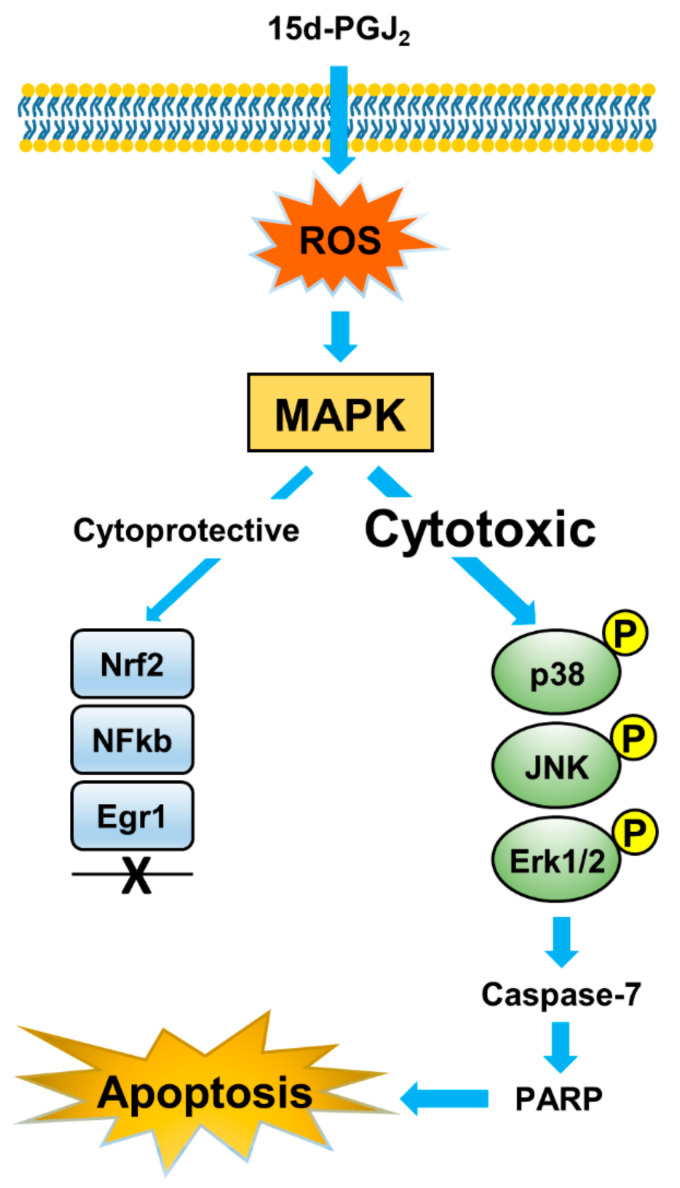
Model depicting the regulation of 15d-PGJ_2_-based apoptosis in OS cells. 15d-PGJ_2_ induces intracellular ROS production, thus activating MAPK-dependent apoptosis. Cytoprotective mechanism are transiently induced by 15d-PGJ_2_; however, they are quickly overwhelmed by apoptosis activation.

To span the bridge between the in vitro and in vivo OS cellular models, we performed ex ovo CAM assay with both U2-OS and Saos-2 cell lines. For comparative purposes, MG-63 cells have been also included. Vehicle- or 15d-PGJ_2_-treated cells were grafted onto the CAM and tumor area, proliferation, and morphology was monitored. We observed substantial tumor growth inhibition in tumors developed from all three OS cell lines (U2-OS (A-B), Saos-2 (G-H), and MG-63 (M-N)) treated with 15d-PGJ_2_ (Figure 5A,B). Not only was the tumor size was smaller, but the density of tumor tissue was even more decreased upon the 15d-PGJ_2_ treatment, as shown with the HE staining for U2-OS (Figure 5A(a,b)), Saos-2 (Figure 5A(i,j)), and MG-63 (Figure 5A(o,p)) onplants. Quantification revealed that significantly smaller tumors formed from 15d-PGJ_2_ pre-treated U2-OS (57%, *p* = 0.0004), Saos-2 (38%, *p* = 0.035), and MG-63 (35%, *p* = 0.0015) cells compared to vehicle-pretreated cells (Figure 5B). We further analyzed the expression of the proliferation marker Ki-67 for U2-OS (Figure 5A(e,f)), Saos-2 (Figure 5A(k,l)), and MG-63 (Figure 5A(q,r)) onplants, which was significantly stronger in control, untreated tumors, indicating reduced cell proliferation of grafted 15d-PGJ_2_-treated OS cells. These results were comparable for all three OS cells. Possible model depicting the regulation of 15d-PGJ_2_-based activation of apoptosis in OS cells is shown in Figure 6.

## 3. Discussion

OS is the most common tumor of bone [1,2]. Due to the late diagnosis and frequent formation of metastases, the establishment of an efficient pharmacological treatment is crucial. 15d-PGJ_2_ is a naturally occurring cyclopentenone PG in the human body [29], which can provide anti-inflammatory [30,31] and anti-angiogenic [30,32], but also pro-apoptotic [17,22,23,24] and anti-metastatic [33] properties in a variety of tissues and cell types. In this study, we aimed to elucidate acute effects and mechanisms of 15d-PGJ_2_ in different OS cell lines.

Previous studies in OS cells have shown inconclusive results suggesting the activation of pro-apoptotic and pro-survival mechanisms as a response to the treatment with 15d-PGJ_2_ [25,26]. 15d-PGJ_2_ increases ROS formation as one of the initial reactions to its anti-inflammatory and anti-tumor effects [26,29,34,35,36,37]. In this study, we found rapid activation of ROS formation upon 15d-PGJ_2_ treatment of OS cells, followed by swift activation of MAPKs already within 5 to 15 min upon treatment of OS cells with 15d-PGJ_2_. Due to the potential dual role of a MAPK activation [38,39], we focused on a selection of proteins that are commonly increased when an intercellular survival mechanism is initiated [34,40,41,42]. In an earlier study, we could show that 15d-PGJ_2_ causes intracellular redox imbalance that further promotes both death and survival pathways in MG-63 OS cells [25]. The results of the present study revealed 15d-PGJ_2_-mediated ROS generation as an early event of a hierarchical series of cell fate decisions that culminate in OS cell apoptosis. Along this line, it is important to note that trabectedin, a semi-synthetic alkaloid originally isolated from the sea squirt *Ecteinascidia Tunicata*, in combination with irinotecan inhibits the growth of orthotopic osteosarcoma xenografts in mice that were established with pelvic OS biopsy material obtained from a 14-year-old patient [43]. A dose-escalation and dose-expansion study suggested that trabectedin i.v. in combination with the orally available PARP inhibitor olaparib shows manageable toxicity at active dose levels and promising preliminary data on antitumor potential in patients with bone and soft-tissue sarcomas [44,45]. In breast cancer and multiple myeloma cells trabectedin increased the generation of ROS and induced apoptosis [46] reminiscent of 15d-PGJ_2_-induced pathways observed during the present study.

Upon transient activation of pro-survival enzymes (Akt, Nrf2, NF-κB, and Egr1) their expression rapidly decreased to or below baseline levels. In accordance with our findings, Shin and colleagues reported rapid inactivation and degradation of Akt in 15d-PGJ_2_-treated leukemia cells that correlated with increased apoptosis susceptibility [29]. We observed a decrease in overall Nrf2 levels associated with increased apoptosis, compatible with findings reported for cisplatin-resistant MG-63 and Saos-2 cells [47]. Thus, we conclude that 15d-PGJ_2_-mediated activation of cytoprotective mechanism is quickly overcome by inhibition of cell proliferation and induction of apoptosis. This suggests that 15d-PGJ_2_ primarily acts as initiator of apoptosis in OS cell lines. Therefore, we suggest that in U2-OS and Saos-2 cells, the cytoprotective mechanisms are less pronounced compared to MG-63 cells [25], which might be dependent on the different genetic background of cells. Both U2-OS and Saos-2 cells are known for higher invasion and motility versus low-invasive MG-63 cells. In contrast, MG-63 cells display higher clonogenicity as compared to the other two cell lines [48,49,50].

ROS generation is considered a primary event during 15d-PGJ_2_-mediated cell death induction in leukemia and colorectal cells, with cytosolic NADPH oxidase and mitochondria (complex 1 and 3) being the major sources for ROS generation [29]. The use of catalase or EUK-134 (a salen-manganese complex antioxidants mimicking catalase activity) blocked PARP cleavage and significantly reduced the number of apoptotic Annexin V-positive cells [29]. Induction of oxidative stress can lead to the modification of redox-sensitive protein thiols that act as intracellular sensor systems. Within the concept of redox signaling, growing evidence suggests that the modification of specific sensor proteins conveys information on the state of cellular homeostasis. Thioredoxin (Trx) represents one of the prototypic thiol-sensitive redox sensors and plays an important role during the induction of cell death. In this scenario, apoptosis signaling kinase 1 (ASK1) forms an inactive complex with reduced Trx1 that inhibits homophilic interaction of ASK1, which is required for full enzymatic activity. However, oxidation of Trx1 leads to the dissociation of Trx1 from ASK1, which induces full kinase activity [51]. As a MAP3 kinase, ASK1 can then initiate downstream activation of p38 and JNK, both promoting the apoptotic machinery leading to cell death [52]. 15d-PGJ_2_, as an electrophile, is able to modify Trx thiols (Cys-35 and Cys-39) via Michael adduct formation [53]. In this study, the cleavage of both PARP and procaspase-7 was not observed in dh-15d-PGJ_2_-treated cells, indicating that the electrophilic C9 in 15d-PGJ_2_ is a definitive moiety to induce OS cell apoptosis, a process most probably mediated via de-repression of the ASK1 signalosome and subsequent activation of the proapoptotic MAPK members p38 and JNK.

As a limitation of our study, we presently cannot exclude the influence of genetic background of different OS cells on effects caused by 15d-PGJ_2_, since MG-63 cells express functional Rb but not p53, U2-OS cells express both functional Rb and p53, while Saos-2 cells lack both Rb and p53 expression. Both, Rb and p53 play pivotal roles in cell fate decisions including cell cycle arrest, DNA damage response and apoptosis [54].

As a strength of our study, we analyzed some basic effects of 15d-PGJ_2_ on non-malignant human osteoblasts expressing both intact p53 and Rb protein. Since our two model cell lines are both of osteoblastic origin [55,56], we considered the human osteoblast cell line hFOB1.19 as a suitable model. Here, we show that non-malignant osteoblasts are characterized by higher resistance to 15d-PGJ_2_, this supporting the role of 15d-PGJ_2_ as a potential therapeutic agent.

15d-PGJ_2_-based generation of ROS promotes p38 MAPK activation and subsequent Akt phosphorylation in MG-63 OS cells [25]. Here, we show that these mechanisms are of importance in different OS cell lines. Caused by 15d-PGJ_2_, cells produce ROS, which in turn phosphorylate MAPKs that in turn may activate downstream MAPK kinases. The p38- and JNK-MAPK pathways in turn activate caspases for the successful completion of the apoptotic pathway. Therefore, the time-dependent increase in expression of the activated forms of these enzymes indicates a successful induction of apoptosis by 15d-PGJ_2_.

The CAM assay is an easily accessed system for studying the growth capability of grafted cells [48] and a suitable tool for preclinical screening of anticancer drugs. It can be much more easily and quickly established than animal models, it is relatively cheap, and does not need ethic committee approval [57]. A previous study, using a panel of eight different OS cell lines to induce tumor formation in the CAM assay, found medium tumor growth for U2-OS and Saos-2 cell lines but only moderate tumor growth with MG-63 cells [57]. We observed efficient formation of solid tumors using U2-OS, Saos-2, and MG-63 cells. The reasons for this discrepancy might be due to (i) differences in growth of onplanted OS cells between in ovo [57] and our ex ovo CAM models, and (ii) the number of onplanted cells which is different in both studies. Most importantly, our data show efficient growth inhibition in tumor onplants from 15d-PGJ_2_-pretreated OS cells. Both the tumor size and the density of tumor tissue was diminished upon cell treatment with 15d-PGJ_2_. As a method bridging the gap between in vitro and in vivo, the CAM model efficiently display pronounced cytotoxic effects of 15d-PGJ_2_ on OS cells, thus further identifying 15d-PGJ_2_ as a promising therapeutic agent against OS.

In conclusion, we found that 15d-PGJ_2_ has pronounced anti-tumorigenic effects on different OS cell lines. It activates increased ROS production, which further triggers a very rapid activation of apoptosis and inhibition of cell proliferation. These effects are more pronounced in malignant OS cells compared to non-malignant osteoblasts. In an ex vivo CAM assay, 15d-PGJ_2_ strongly attenuates OS tumor formation. Altogether, our results suggest 15d-PGJ_2_ as a very potent naturally occurring substance for treatment of osteosarcoma and facilitate further in vivo/clinical studies in this field.

## 4. Materials and Methods

### 4.1. Materials

15d-PGJ_2_ (15-deoxy-Δ^12,14^-prostaglandin J_2_, #18570, Cayman Chemical Company, Ann Arbor, MI, USA) was dissolved under sterile conditions in dimethyl sulfoxide (DMSO, Sigma-Aldrich, MO, USA). Aliquots (20 mM) were stored at −20 °C and used within two weeks. If not otherwise stated, DMSO has been used as vehicle control at a concentration corresponding to 20 µM 15d-PGJ_2_ (0.04%, *v*/*v*). All cell culture media and supplements were from Gibco (Thermo Fisher Scientific, Schwerte, Germany). Fetal calf serum (FCS, Thermo Fisher Scientific) was heat-inactivated in house (56 °C for 30 min).

### 4.2. Cell Culture

OS cell lines (U2-OS (HTB-06), Saos-2 (HTB-85), MG-63 (CRL-1427)) were of human origin (ATCC, Manassas, VA, USA). U2-OS and MG-63 cells were cultured in Eagle’s Alpha-Minimal Essential medium (EMEM) supplemented with 2.5 mM (*w*/*v*) L-glutamine, 10% (*v*/*v*) FCS, and 1% (*v*/*v*) penicillin/streptomycin (Gibco). Saos-2 cells were cultured in McCoy 5A medium (Lonza, Switzerland) supplemented with 2.5 mM (*w*/*v*) L-glutamine, 10% (*v*/*v*) FCS, and 1% (*v*/*v*) penicillin/streptomycin. OS cell lines were cultivated under standardized conditions (5% CO_2_, 37 °C, 98% humidity) and medium was exchanged twice per week. Human osteoblasts (hFOB1.19), provided by the Core Facility Alternative Biomodels & Preclinical Imaging of the Medical University Graz, were cultivated at 34 °C in a 1:1 (*v*/*v*) mixture of Ham’s F12 medium and Dulbecco’s Modified Eagle’s Medium (DMEM) supplemented with 2.5 mM (*w*/*v*) L-glutamine, 10% (*v*/*v*) FCS, 1% (*v*/*v*) penicillin/streptomycin, and 0.3 mg/mL G-418 (Geneticin, InvivoGen, Toulouse, France). All cell lines were split at 80–90% confluence and cell numbers were determined by CASY (OMNI Life Sciences, Bremen, Germany). The mycoplasma test was performed every three months.

### 4.3. Proliferation and Viability Assay (MTT)

Cells were treated in 12-well plates (180,000 cells/well) with 20 μM 15d-PGJ_2_, a suitable concentration reported previously in numerous studies [29,36,58,59,60,61], for indicated time periods (4, 18, 24 h) prior to a 30 min incubation with MTT (3-(4,5-dimethylthiazol-2-yl)-2,5-diphenyltetrazolium bromide) (0.5 mg/mL; dissolved in serum-free medium). Afterwards, the resulting insoluble dye *formazan* was made soluble by addition of 350 μL acidic isopropanol (0.04 M HCl in isopropanol). Samples were then further analyzed via optical measurement (with the emission/correction wavelengths of 570/630 nm) by using a microtiter plate reader (BMG Labtech, Ortenberg, Germany). The results were compared to the vehicle control (0.04%) and assessed by appropriate statistics.

### 4.4. Wound Healing Assay

Wound healing assay was performed in order to investigate the influence of 15d-PGJ_2_ on cell proliferation and motility. Cells were seeded in cell culture inserts (Ibidi GmbH, Graefelfing, Germany) places in a 12-well plates and grown until approx. 90% confluence prior to incubation with 15d-PGJ_2_ for 24 h. Afterwards, cell culture inserts were removed, leaving a defined 500 µm cell-free gap, and medium was changed to remove detached cells. Cells treated with DMSO (0.04%) were used as a vehicle control. Cell were then incubated at standardized conditions and photos were taken at time points 0, 24, 36, and 48 h (Nikon ECLIPSE Ts2, 40× magnification). Gap closure was measured and quantified by Fiji ImageJ Wound Healing plug-in (open source) and calculated as percentage of the open gap area compared to the zero time point.

### 4.5. Colony Formation Assay

Cells were seeded in 6-well plates and treated with 20 µM 15d-PGJ_2_ or corresponding vehicle concentration (DMSO, 0.04%) for 24 h. Afterwards, cells were trypsinized, counted, and the appropriate cell number (300 for U2-OS cells, 1000 for Saos-2 cells) was seeded into 6-well plates and incubated for additional 10 days (U2-OS) or 15 days (Saos-1). On the last day, colonies were fixed (methanol:glacial acetic acid; 3:1 (*v*/*v*)) and stained with 0.4% (*w*/*v*) aqueous crystal violet solution (C6158, Sigma). Colonies were then scanned on a white background and counting was performed by ImageJ with Colony Counter plug-in. Only colonies containing at least 50 cells were taken into account.

### 4.6. Intracellular ROS Measurement

Intracellular redox homeostasis was assessed as previously described by our group [25]. Briefly, U2-OS and Saos-2 cells were grown until a confluence of approx. 80% in 24-well plates and treated with 20 μM 15d-PGJ_2_. Cells were incubated with the ROS-reactive dye carboxy-H_2_DCFDA (5-(and -6)-carboxy-2′,7′-dichlorodihydrofluorescein diacetate, Invitrogen) in phosphate-buffered saline (PBS) for 30 min at 37 °C. Afterwards, the cells were washed twice with ice-cold PBS and lysed with 300 μL of 3% (*v*/*v*) Triton X-100 in PBS for 30 min, followed by a 15 min incubation in 50 μL absolute ethanol with shaking (1350 rpm) at 4 °C. Finally, samples were centrifuged at 10,000 rpm at 4 °C to remove cell debris, and in supernatants the DCF (2′,7′-dichlorofluorescein) was measured at emission/correction wavelengths of 485/540 nm, respectively. A microtiter plate reader (CLARIOstar, BMG Labtech, Ortenberg, Germany) was used to measure fluorescence intensities.

### 4.7. Immunoblot Analysis

Treated cells were harvested and lysed in RIPA buffer (Sigma, R0278) containing protease inhibitor cocktail (A32953, Thermo Scientific) and phosphatase inhibitors (A32957, Thermo Scientific). Upon sonication (2 × 5 s), soluble total proteins were separated by centrifugation at 13,000 rpm for 10 min. Supernatants were collected total protein concentration was measured using BCA protein assay kit (Thermo Fisher Scientific). Protein extracts (15 µg) were denatured for 10 min at 95 °C, separated via electrophoresis on polyacrylamide gel, and transferred onto nitrocellulose or PVDF membranes (0.45 µm, Amersham). The membranes were blocked with 5% (*w*/*v*) non-fat milk or bovine serum albumin (BSA) for 1 h at room temperature (RT, 25 °C), followed by an overnight incubation at 4 °C with the primary antibody. All antibodies and dilutions used in this study are summarized in Table 1. Afterwards, the membranes were washed in TBST three times for 10 min and incubated with appropriate secondary antibodies linked with horseradish peroxidase (HRP). Final development was performed with ECL Prime Western Blot Detection Reagents (Amersham) with signal detection on Chemi-Doc Touch Biorad device. Membranes were stripped with the Restore Plus Western blot stripping buffer (Thermo Fisher Scientific). For loading control, antibody raised against alpha tubulin (α-tubulin) was used. Intensity normalization to the loading control (α-tubulin) was performed by using Image Lab software 6.0.1 (Biorad).

### 4.8. Annexine V/propidium Idodide (PI) Staining

After reaching approx. 80% confluence, U2-OS and Saos-2 cells were treated with 20 μM 15d-PGJ_2_ for 12, 18 and 24 h. Cells were stained by FITC Annexin V Apoptosis Detection Kit 1 (BD Biosciences), followed by a wash in cold PBS, and a 15 min incubation at RT with a 100 μL of 1 × binding buffer (5 μL of Annexin V FITC and 5 μL of PI) in the dark. The results were obtained by cytometric analysis on the Guava EasyCyte 8 (Millipore) and analyzed by using the InCyte 3.1 software (Millipore).

### 4.9. Ex Ovo Avian CAM Assay

Chorioallantoic membrane (CAM) assay was carried out using the ex ovo CAM method according to Deryugina and Quigley [62]. Briefly, fertilized white Lohmann chicken eggs were cleaned and incubated at 37.5 °C and 60% humidity (Incubator Easy 200, J. Hemel Brutgeraete, Verl, Germany). On day three of embryonic development, the egg shell was cracked in a sterile weigh boat and incubated at 37.5 °C and 60% humidity for further 6 days. On day nine of embryonic development, treated OS cells were grafted on CAM: OS cell lines (U2-OS, Saos-2, and MG-63) were grown until a confluence of approx. 80%, treated with 20 μM 15d-PGJ_2_ or DMSO for 24 h, and afterwards gently harvested by trypsinization. Silicone rings (Ø5 mm) were carefully placed between embryonic blood vessels, and 15d-PGJ_2_-treated or control (DMSO) OS cell suspension was added into silicon rings (1 × 10^6^ cells/onplant). The embryos were incubated again for four days and afterwards the onplants were harvested, fixed in 4% paraformaldehyde (PFA), dehydrated and embedded in paraffin. The paraffin tissue blocks were then cut into 7 µm thick sections and fixed on glass tissue slides before immunohistochemical staining for the proliferation marker Ki-67.

### 4.10. Immunohistochemistry

Immunohistochemical staining for Ki-67 was performed in the automated system DAKO OMNIS with primary DAKO anti Ki-67 antibody Clone MIB-1 (GA62661-2) and DAKO OMNIS Flex HRP detection system. All slides were documented with the Nikon Eclipse E400 microscope and Zwo Asi 183 MC pro camera. Immunohistochemical staining was evaluated by two experienced histopathologists (MA, NGTW).

### 4.11. Statistical Calculations

All statistical calculations were performed by GraphPad Prism (v. 5.0). All values are represented as mean ± SD of at least three independent experiments. A two-sided student’s *t*-test was used to determine the level of statistical differences. A value of *p* ≤ 0.05 was considered statistically significant (*).

## Figures and Tables

**Table 1:** Primary antibodies and dilutions used in this study.

Antibody	Company	Cat. Nr.	Host	Dilution
pJNK	Cell Signaling	#4668	rabbit	1:1000
JNK	Cell Signaling	#9252	rabbit	1:2000
pp38	Cell Signaling	#9211	rabbit	1:1000
p38	Cell Signaling	#9212	rabbit	1:2000
p-Erk1/2	Cell Signaling	#9106	mouse	1:1000
Erk1/2	Cell Signaling	#9102	rabbit	1:2000
p-Akt (Ser473)	Cell Signaling	#9271	rabbit	1:1000
Akt	Cell Signaling	#9272	rabbit	1:2000
NF-κB (p56)	Cell Signaling	#8242	rabbit	1:1000
Egr1	Cell Signaling	#4154	rabbit	1:1000
Nrf2	R&D Systems	#MAB3925	mouse	1:500
PARP	Cell Signaling	#9542	rabbit	1:1000
Cl. Caspase-7	Cell Signaling	#9491	rabbit	1:1000
Alpha-tubulin	Cell Signaling	#2125	rabbit	1:2000

## Data Availability

All data generated and analyzed during this study are available from corresponding author on reasonable request.

## Abbreviations

OS	Osteosarcoma
PG	Prostaglandin
MAPK	Mitogen-activated protein kinase
ROS	Reactive oxygen species
CAM	Chorioallantoic membrane
DMSO	Dimethyl sulfoxide
EMEM	Eagle’s minimum essential medium
DMEM	Dulbecco’s Modified Eagle’s *Medium*
FCS	Fetal calf serum
MTT	3-(4,5-dimethylthiazol-2-yl)-2,5-diphenyltetrazolium bromide
H_2_DCFDA	5-(and -6)-carboxy-2′,7′-dichlorodihydrofluorescein diacetate
PBS	Phosphate-buffered saline
DCF	Dichlorofluorescein
BCA	Bicinchoninic acid assay
PVDF	Polyvinylidene fluoride
BSA	Bovine serum albumine
TBST	Tris-buffered saline with Tween 20
HRP	Horseradish peroxidase
RT	Room temperature
PFA	Paraformaldehyde
SD	Standard deviation
ERK1/2	Extracellular signal-regulated kinase
NAC	N-acetyl-cystein
PDTC	Pyrrolidine dithiocarbamate
SOD	Superoxide dismutase
PARP	Poly(ADP-ribose)-polymerase
JNK	c-Jun N-terminal kinase
AKT	Protein kinase-B
Nrf2	Nuclear factor E2-related factor 2
Egr1	Early growth response factor 1
NF-κB	Nuclear factor NF-kappa B
Trx	Thioredoxin
ASK1	Apoptosis signaling kinase 1
Rb	Retinoblastoma
